# *Pseudomonas aeruginosa* increases the susceptibility of *Candida albicans* to amphotericin B in dual-species biofilms

**DOI:** 10.1093/jac/dkad228

**Published:** 2023-07-31

**Authors:** Farhana Alam, Sarah A Blackburn, Jack Davis, Keely Massar, Joao Correia, Hung-Ji Tsai, Jessica M A Blair, Rebecca A Hall

**Affiliations:** Institute of Microbiology and Infection, School of Biosciences, University of Birmingham, Birmingham, B15 2TT, UK; Kent Fungal Group, Division of Natural Sciences, School of Biosciences, University of Kent, Canterbury, CT2 7NJ, UK; Kent Fungal Group, Division of Natural Sciences, School of Biosciences, University of Kent, Canterbury, CT2 7NJ, UK; Kent Fungal Group, Division of Natural Sciences, School of Biosciences, University of Kent, Canterbury, CT2 7NJ, UK; Institute of Microbiology and Infection, School of Biosciences, University of Birmingham, Birmingham, B15 2TT, UK; Institute of Microbiology and Infection, School of Biosciences, University of Birmingham, Birmingham, B15 2TT, UK; Institute of Microbiology and Infection, College of Medical and Dental Sciences, University of Birmingham, Birmingham, B15 2TT, UK; Kent Fungal Group, Division of Natural Sciences, School of Biosciences, University of Kent, Canterbury, CT2 7NJ, UK

## Abstract

**Background:**

Biofilms are the leading cause of nosocomial infections and are hard to eradicate due to their inherent antimicrobial resistance. *Candida albicans* is the leading cause of nosocomial fungal infections and is frequently co-isolated with the bacterium *Pseudomonas aeruginosa* from biofilms in the cystic fibrosis lung and severe burn wounds. The presence of *C. albicans* in multispecies biofilms is associated with enhanced antibacterial resistance, which is largely mediated through fungal extracellular carbohydrates sequestering the antibiotics. However, significantly less is known regarding the impact of polymicrobial biofilms on antifungal resistance.

**Results:**

Here we show that, in dual-species biofilms, *P. aeruginosa* enhances the susceptibility of *C. albicans* to amphotericin B, an effect that was biofilm specific. Transcriptional analysis combined with gene ontology enrichment analysis identified several *C. albicans* processes associated with oxidative stress to be differentially regulated in dual-species biofilms, suggesting that *P. aeruginosa* exerts oxidative stress on *C. albicans*, likely through the secretion of phenazines. However, the mitochondrial superoxide dismutase *SOD2* was significantly down-regulated in the presence of *P. aeruginosa*. Monospecies biofilms of the *sod2Δ* mutant were more susceptible to amphotericin B, and the susceptibility of these biofilms was further enhanced by exogenous phenazines.

**Conclusions:**

We propose that in dual-species biofilms, *P. aeruginosa* simultaneously induces mitochondrial oxidative stress, while down-regulating key detoxification enzymes, which prevents *C. albicans* mounting an appropriate oxidative stress response to amphotericin B, leading to fungal cell death. This work highlights the importance of understanding the impact of polymicrobial interactions on antimicrobial susceptibility.

## Introduction

*Candida albicans* is a commensal and opportunistic fungal pathogen of humans that is frequently co-isolated from infection sites with the Gram-negative bacterium *Pseudomonas aeruginosa*.^1^ Extensive research has shown that these two microbes undergo a complex range of interactions, the outcome of which appears to be dependent on the surrounding environment. For example, *P. aeruginosa* has been shown *in vitro* to bind and kill *C. albicans* hyphae,^2^ whereas *in vivo*, *P. aeruginosa* and *C. albicans* have been shown to have an agonistic relationship, resulting in enhanced pathogenesis.^3^*P. aeruginosa* secretes a broad range of virulence factors including phenazines, siderophores, haemolysins and phosphatases, which are regulated by one or more quorum-sensing systems. Several of these secreted products have been shown to directly affect *C. albicans*, with the phenazines resulting in the induction of reactive oxygen species (ROS)-induced stress, and inhibition of fungal growth and morphogenesis,^4,5^ whereas siderophores impose nutrient restriction. On the other hand, *C. albicans* secretes the quorum-sensing molecule farnesol, which inhibits the *Pseudomonas* quinolone signal pathway,^6^ and imposes ROS-induced stress on other microbes.^7,8^

*C. albicans* and *P. aeruginosa* are most commonly co-isolated from the lungs of cystic fibrosis patients,^9^ and from wounds of burn victims,^10^ where the two microbes tend to grow together in biofilms. Biofilms are communities of cells encased in an extracellular matrix (ECM), which provides protection from the host’s immune responses, and increases the resistance of microbes to antimicrobial agents. Cells in a monospecies biofilm can exhibit MIC values 100–1000-fold greater than their planktonic counterparts. However, in mixed-species biofilms, the microbes interact through secreted signalling molecules, direct cell–cell interactions and through nutrient competition,^11^ all of which have the potential to affect antimicrobial resistance. Furthermore, each species contributes specific extracellular polysaccharides to the ECM, and fungi produce hyphae that act as molecular scaffolds that together alter the structure and composition of the biofilm. These interactions affect the susceptibility of the microbes to antimicrobial therapy. For example, the presence of *C. albicans* in bacterial biofilms promotes the resistance of *Staphylococcus aureus*, *P. aeruginosa* and *Escherichia coli* to a variety of antibiotics.^12–15^ Although the precise molecular mechanisms behind this enhanced antibacterial resistance are not fully understood, it is thought that the contribution of fungal extracellular polysaccharides to the ECM sequesters the antibiotic molecules and limits their diffusion through the biofilm. Furthermore, microbe–microbe interactions and nutrient competition may alter the transcriptional profile of bacterial cells, priming the cells to be more tolerant to the antibiotic. However, how these microbe–microbe interactions affect the response to antifungal treatment is unknown.

Currently there are only a handful of classes of antifungal drugs on the market, the most widely used of which are the azoles (fluconazole, miconazole, etc.), which target ergosterol biosynthesis; the echinocandins (caspofungin and anidulafungin), which target the synthesis of β-glucan; and the polyenes (amphotericin B and nystatin), which target ergosterol. The widespread use of antifungals in both hospital and agricultural settings has resulted in the rapid rise of antifungal resistance, with the WHO identifying MDR *Candida* infections as a major cause for concern. With limited new antifungal drugs in the pipeline, there is an urgent need to preserve and improve the efficacy of the available antifungals. Here, we demonstrate that in a dual-species biofilm *P. aeruginosa* enhanced the susceptibility of *C. albicans* to the antifungal amphotericin B but had minimal effect on the susceptibility of the fungus to fluconazole or caspofungin. The enhanced susceptibility of *C. albicans* to amphotericin B was mediated by the *P. aeruginosa*-dependent repression of the fungal superoxide dismutase, *SOD2,* resulting in *C. albicans* being exposed to enhanced reactive oxygen stress.

## Methods

### Strains and media

*C. albicans* strains were maintained on YPD (yeast peptone dextrose) agar and grown in YPD broth, whereas *P. aeruginosa* strains were grown and maintained on LB medium. All biofilm assays were performed in Mueller–Hinton broth (MHB). The strains used in this study are listed in Table S1 (available as Supplementary data at *JAC* Online).

### Biofilm assay

Biofilm assays were adapted from previously described methods.^15^ In brief, *C. albicans* was grown overnight in YPD and *P. aeruginosa* was grown overnight in LB, and cultures were washed in PBS. *C. albicans* strains were resuspended at 1 × 10^6^ cells/mL and *P. aeruginosa* strains were resuspended to an OD_600_ of 0.2 in MHB. Each well of 96-well plates contained 100 μL (1 ×10^5^ cells) *C. albicans* and 10 μL (2.5 ×10^6^ cells) of *P. aeruginosa* (final ratio fungus:bacterium = 1:25). Plates were incubated at 37°C for 2 h to allow cells to adhere, at which point the medium was replaced with fresh sterile medium, and plates incubated statically at 37°C for 24 h. Cells not part of the biofilm were removed and medium replaced with fresh MHB containing the appropriate concentration of antifungal agent, and plates incubated for 2 h or 18 h. Medium was replaced with 100 μL PBS containing 50 μg/mL DNase I and plates were incubated at 37°C for 1 h to degrade the ECM. Biofilms were detached from the plates by scraping, serially diluted, and plated onto YPD agar supplemented with 100 μg/mL tetracycline to determine *C. albicans* cfus, or cetrimide agar to quantify *P. aeruginosa* cfus.

To assess the effects of phenazines or H_2_O_2_ on antifungal resistance, compounds were added to single-species biofilms at the indicated concentrations, after the adherence step, and biofilms formed as outlined above.

To assess the effects of dead *P. aeruginosa* on antifungal resistance, overnight bacterial cultures were washed in PBS and cells inactivated by either heat-killing at 100°C in PBS for 1 h, or fixed in 4% paraformaldehyde (PFA) for 1 h at room temperature. Inactivated cells were washed with PBS and resuspended in MHB to an OD_600_ of 0.8 to mimic the *P. aeruginosa* cfus recovered from mature biofilms. Biofilms were then established and quantified as described above.

To assess the impact of biofilm supernatants on the susceptibility of *C. albicans* to amphotericin B, supernatants from multiple 24 h biofilms were pooled, filter sterilized, snap frozen and stored at −20°C for a maximum of 6 days. Single-species biofilms were then established in MHB containing 50% biofilm supernatant.

### RNA sequencing

Biofilms were formed as described previously.^16^*C. albicans* (SC5314) was grown overnight in YPD at 37°, 200 rpm and *P. aeruginosa* (PAO1) was grown overnight at 37°C, 200 rpm in LB, respectively. Cultures were washed with PBS; *C. albicans* was resuspended to 1 ×10^6^ cells/mL and *P. aeruginosa* was resuspended to an OD_600_ of 0.2 in MHB. Biofilms were grown in six-well plates, containing 3 mL *C. albicans* (3 ×10^6^ cells) and 300 μL (75 × 10^6^ cells) of *P. aeruginosa* (final ratio 1:25). Plates were incubated at 37°C for 2 h to allow cells to adhere, after which non-adhered cells were removed and replaced with 3 mL of fresh MHB. Plates were incubated at 37°C for 24 h, then medium was replaced with fresh MHB, and biofilms incubated for a further 4 h. Medium was replaced with 2 mL of PBS containing 50 μg/mL DNase I and incubated at 37°C for 1 h to degrade extracellular DNA. Biofilms were detached from the plate by scraping, triplicate biofilms were then pooled, and 50 μL was serially diluted and plated on cetrimide agar or YPD supplemented with 100 μg/mL tetracycline to check for contamination. Remaining biofilm cells were centrifuged at 3500 rpm at 4°C for 5 min, and pellets snap frozen in liquid nitrogen. Four biological replicates were shipped to GeneWiz^®^, UK, for RNA extraction, sequencing and basic bioinformatic analysis.

### RNA extraction and sequencing

RNA was extracted from the biofilms by GeneWiz^®^ using the Qiagen RNeasy Plus mini kit. Library preparation was done in the following stages: (i) ribosomal RNA depletion; (ii) RNA fragmentation and random priming; (iii) first- and second-strand cDNA synthesis; (iv) end repair, 5′ phosphorylation and dA-tailing; and (v) adapter ligation, PCR enrichment and sequencing. Paired-end sequencing was performed using Illumina HiSeq 4000 (2 × 150 bp configuration, single index, per lane).

### Bioinformatic analysis

Sequence quality of each sample was evaluated by determination of the number of reads, the yield (Mbases), the mean quality score and the percentage of reads over 30 bases in length. FastQC software was used to determine per base sequence quality and per sequence GC content. Sequence reads were trimmed to remove adapter sequences and nucleotides with poor quality, using Trimmomatic v.0.36. The trimmed reads were mapped to the *C. albicans* reference genome, available on ENSEMBL, using the STAR aligner v.2.5.2b. For dual-species biofilms, reads were first mapped to the *P. aeruginosa* PAO1 genome available on ENSEMBL, and then unmapped reads were aligned to the *C. albicans* genome. Unique gene hit counts were calculated using featureCounts from the Subread package v.1.5.2. Only unique reads that fell within exonic regions were counted (the maximum hits per read was set to 10 by default). One biological replicate of the *C. albicans* monospecies biofilms (CA 0M-6) had a significant number of reads that did not align to the *C. albicans* genome or the *P. aeruginosa* genome and was therefore excluded from downstream analysis. Differential gene expression analysis was performed using DESeq2. Principal component analysis (PCA) was performed to reveal the similarities within and between groups, with PCA plots included in the output (Figure S1). The DESeq2 output file (*C. albicans*_expression_CA_vs_PACA.xlsx) and the raw and normalized reads and transcript per million (TPM) values for all *C. albicans* (*C_albicans*_counts.xlsx) and *P. aeruginosa* (*P_aeruginosa*_count_data.xlsx) genes are available at the Gene Expression Omnibus (GEO) database (https://www.ncbi.nlm.nih.gov/geo/), under accession number GSE167137.

To reveal the global genomic responses, defined differentially expressed genes were compared with genes under oxidative stress, heat-shock stress and hyperosmotic stress with different timings, 0, 10, 30 and 60 min,^17^ respectively, using Pearson correlation coefficient in custom R scripts (cor.test).

### Enrichment analysis

For *C. albicans* transcriptomic analysis, differential expression of genes between conditions was considered significant if the adjusted *P* value (*P*adj) was ≤0.05 and the log_2_-fold change was ≥1 or −1.^18–20^ Gene ontology (GO) analysis was performed using the *Candida* Genome Database (CGD) Gene Ontology Slim Mapper.^21,22^ This tool maps the annotations from each gene list to GO Slim terms, which are broad, high-level GO terms that are specific to the selected *Candida* species—in this case *C. albicans*.^22^ KEGG pathway enrichment analysis was done using KOBAS 3.0 software,^23^ as described previously.^24^

### Quantification of ergosterol

Biofilms were formed as described above. After the 24 h incubation, biofilms were washed with PBS and fixed with 4% PFA for 1 h at room temperature. Biofilms were disrupted and then stained with filipin (0.05 mg/mL) for 2 h at room temperature. Stained biofilm cells were washed with PBS, mounted onto glass microscope slides and imaged on a Zeiss AxioObserver at ×63  magnification, acquiring 30–33 images per sample. To quantify the amount of ergosterol, the median fluorescent intensity of the filipin staining was quantified using FIJI software. Up to five cells per image were outlined, as well as five spherical areas containing no cells (background fluorescence readings). The mean corrected total cell fluorescence (CTCF) was calculated for each image, in which CTCF = integrated density − (area of selected cell × mean fluorescence of background readings). Data were analysed using one-way analysis of variance (ANOVA) and the Holm–Šidák multiple comparisons test.

### Measurement of ROS

To quantify the amount of ROS in the single- and dual-species biofilms, biofilms were grown in triplicate for 24 h, washed with PBS and then stained with 10 μM 2′,7′-dichlorodihydrofluorescein diacetate (H_2_DCFDA) at 37°C for 45 min, before a final wash in PBS. Images were acquired using a Zeiss LSM880/Elyra/Axio Observer.Z1 Confocal Microscope (Carl Zeiss Inc.) with a ×10  objective. A 488 nm wavelength laser was used to excite samples, with consistent laser power and gain maintained throughout, and fluorescent signal between 500 and 550 nm wavelengths captured. ROS staining was quantified through mean fluorescence intensity of three different 3 722.5 μm^2^ regions per technical biofilm replicate, per condition, across three independent biological experiments. Biofilms grown in the presence of 5 mM H_2_O_2_ served as a positive control for ROS production, and background fluorescence was checked against unstained control biofilms. Data were analysed using one-way ANOVA and the Holm–Šidák multiple comparisons test.

## Results

### P. aeruginosa increases the susceptibility of C. albicans to amphotericin B

**Figure 1.**
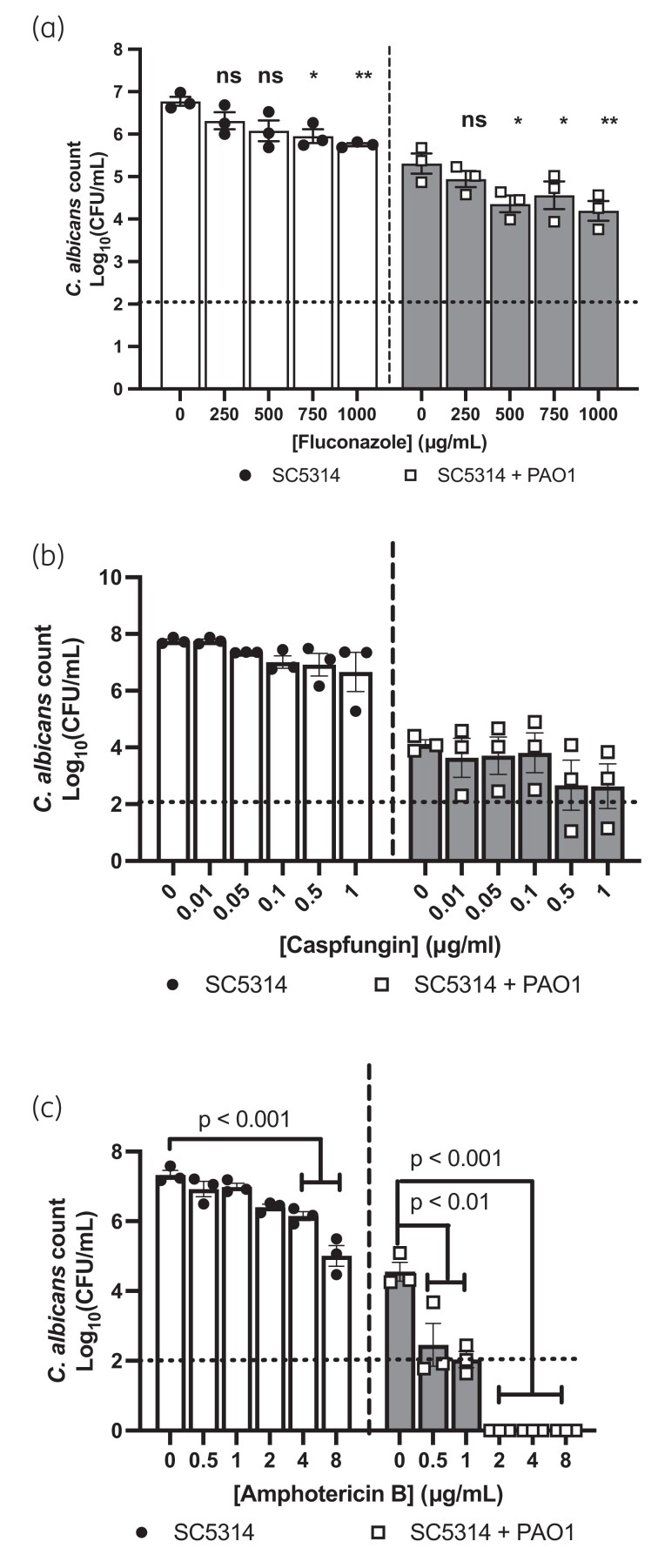
*P. aeruginosa* enhances the susceptibility of *C. albicans* to amphotericin B in dual-species biofilms. Twenty-four-hour preformed single- or dual-species biofilms were treated with increasing concentrations of (a) fluconazole, (b) caspofungin or (c) amphotericin B for 18 h (a and b) or 2 h (c). Data are the log_10_(mean) ± the SEM from three biological replicates. Dotted line represents the minimal level of detection of the assay. Data were analysed using two-way ANOVA and the Holm–Šidák multiple comparisons test (*P < 0.05, **P < 0.01, ns, not significant).

Previously we have shown that dual-species biofilms of *C. albicans* and *P. aeruginosa* have increased tolerance to antibiotics, as a result of the fungal ECM sequestering the drugs.^15^ Therefore, we hypothesized that *P. aeruginosa* may also impact the antifungal susceptibility of *C. albicans*. To test this hypothesis, 24 h mono- or dual-species biofilms were treated with antifungal drugs for either 18 h (fluconazole and caspofungin) or 2 h (amphotericin B) and then *C. albicans* culturability was quantified by plating. The susceptibility of *C. albicans* in dual-species biofilms to fluconazole and caspofungin was not affected compared with the monospecies control (Figure 1a, b). However, treatment of the dual-species biofilms with the polyene amphotericin B resulted in a significant reduction in *C. albicans* cfus, with negligible fungal cells detected when the dual-species biofilms were treated with 2 μg/mL amphotericin B (Figure 1c). On the other hand, treatment of monospecies biofilms with amphotericin B still resulted in significant fungal growth, even when treated with 8 μg/mL amphotericin B. Amphotericin B had no impact on *P. aeruginosa* at any of the concentrations tested, whereas at high concentrations fluconazole reduced the culturability of *P. aeruginosa* (Figure S1a, b). Therefore, these data suggest that the presence of *P. aeruginosa* in the biofilm results in a significant increase in the susceptibility of *C. albicans* to the polyene class of antifungals.

**Figure 2.**
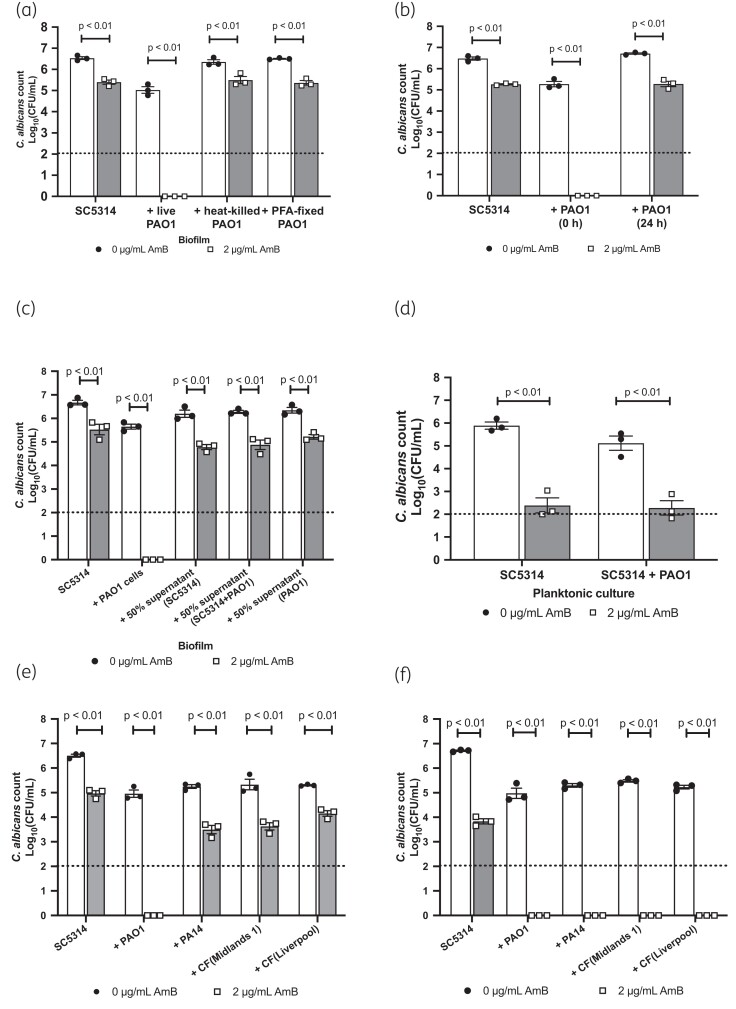
Impact of *P. aeruginosa* on amphotericin B susceptibility is biofilm specific. (a) SC5314 biofilms were grown in the presence of either heat-killed or PFA-fixed *P. aeruginosa* cells for 24 h and then treated with 2 μg/mL amphotericin B for 2 h. (b) SC5314 biofilms were formed with PAO1 cells added at the start (0 h) of biofilm formation or after *C. albicans* had formed a robust biofilm (24 h). (c) SC5314 was grown as single-species biofilms in the presence of 50% sterile supernatant from mature 24 h biofilms. (d) SC5314 was grown planktonically in the presence or absence of PAO1 for 24 h and then treated with 2 μg/mL amphotericin B for 2 h. (e) SC5314 biofilms were formed in the presence of different strains of *P. aeruginosa*, including two clinical isolates from cystic fibrosis patients, and treated with 2 μg/mL amphotericin B for 2 h. (f) SC5314 biofilms were formed in the presence of different strains of *P. aeruginosa*, including two clinical isolates from cystic fibrosis patients, and treated with 2 μg/mL amphotericin B for 18 h. Dotted line represents the minimal level of detection of the assay. Data are the log_10_(mean) ± the SEM from three biological replicates. Data were analysed using two-way ANOVA and the Holm–Šidák multiple comparisons test.

To determine the role of *P. aeruginosa* in this interaction, we grew *C. albicans* biofilms in the presence of heat-killed or fixed *P. aeruginosa* cells and added live *P. aeruginosa* to mature *C. albicans* monospecies biofilms just prior to the addition of amphotericin B. *C. albicans* susceptibility to the polyene was only increased in the presence of live bacteria, which had been directly incorporated into the *C. albicans* biofilm (Figure 2a, b). Furthermore, establishing biofilms in the presence of supernatants from mature biofilms did not affect the susceptibility of *C. albicans* to amphotericin B (Figure 2c), suggesting that bacterial viability and the architecture of the biofilm are important for the observed phenotype. In agreement with this, the susceptibility of *C. albicans* to amphotericin B was not altered when *C. albicans* was co-cultured with *P. aeruginosa* under planktonic conditions (Figure 2d).

To confirm that the impact of *P. aeruginosa* on *C. albicans* amphotericin B susceptibility is a general trait of *P. aeruginosa*, we screened several *P. aeruginosa* clinical isolates for their ability to suppress the resistance of *C. albicans* to the polyene. The viability of *C. albicans* was significantly reduced in all dual-species biofilms treated with amphotericin B, although not as significantly as in the presence of PAO1 (Figure 2e). However, increasing the incubation time of the biofilms with the antifungal from 2 to 18 h killed all fungal cells (Figure 2f), despite *P. aeruginosa* cfu counts remaining consistent (Figure S1d). Therefore, *P. aeruginosa* enhances the susceptibility of *C. albicans* to amphotericin B in dual-species biofilms.

### P. aeruginosa increases the susceptibility of clinical C. albicans isolates and Candida dubliniensis to amphotericin B

**Figure 3.**
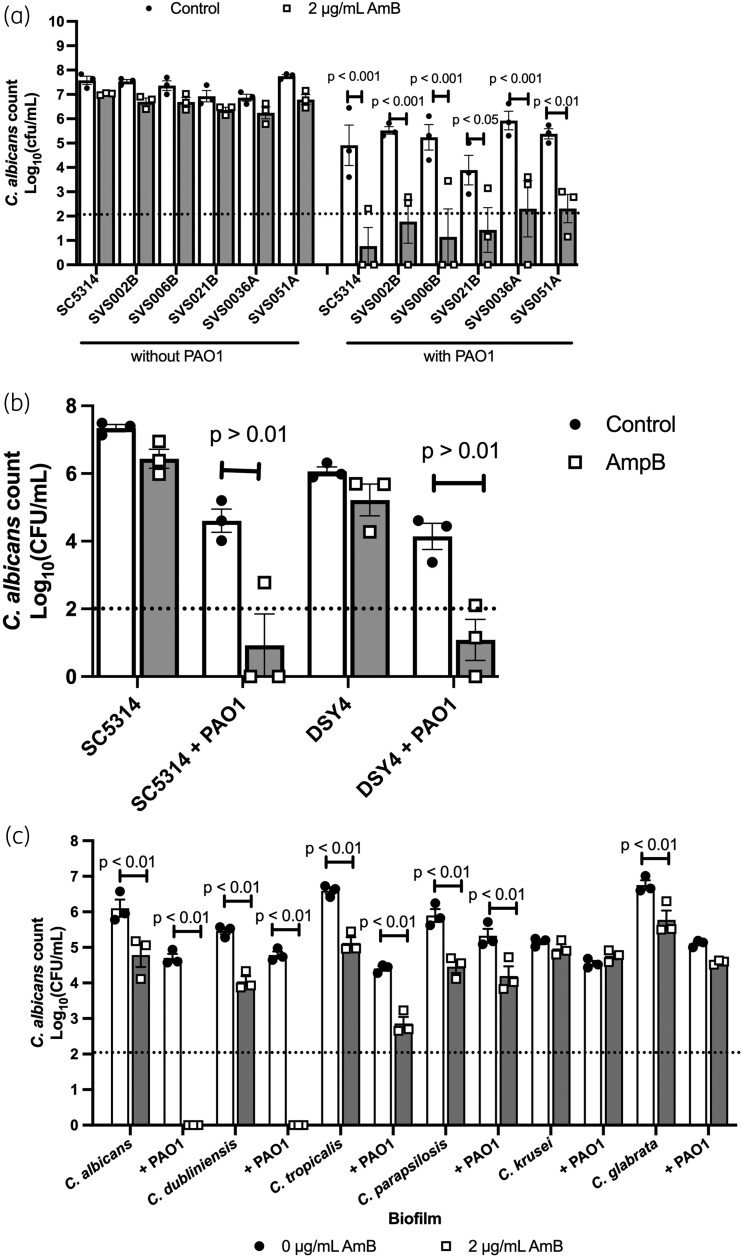
*P. aeruginosa* also increases the susceptibility of *C. albicans* clinical isolates and *C. dubliniensis* to amphotericin B. (a) Clinical isolates of *C. albicans* were grown in single- or dual-species biofilms with PAO1 for 24 h and then biofilms were treated with 2 μg/mL amphotericin B for 2 h. Data are the log_10_(mean) ± the SEM from three biological replicates. (b) The DSY4 strain, which has increased resistance to amphotericin B, was grown in single or dual species biofilms with PAO1 for 24 h and then biofilms were treated with amphotericin B for 2 h. Data are the log_10_(mean) ± the SEM. (c) Medically important *Candida* species were grown in single- or dual-species biofilms with PAO1 for 24 h and then biofilms were treated with 2 μg/mL amphotericin B for 2 h. Data are the log_10_(mean) ± the SEM from three biological replicates. Dotted line represents the minimal level of detection of the assay. Data were analysed using two-way ANOVA and the Holm–Šidák multiple comparisons test.

To confirm that the impact of *P. aeruginosa* on the susceptibility of *C. albicans* to amphotericin B is a general response of *C. albicans*, we tested a series of clinical isolates. All *C. albicans* isolates tested exhibited reduced culturability after treatment with amphotericin B in dual-species biofilms (Figure 3a). Next, to assess whether a similar effect was observed with *C. albicans* strains with increased resistance to amphotericin B, we tested the *erg6* mutant strain DSY4, which has a higher MIC for amphotericin B than SC5314 due to altered sterol composition of the cell membrane, but still displays good growth rates in rich media. In the presence of *P. aeruginosa,* DSY4 was more susceptible to amphotericin B, as observed for SC5314 (Figure 3b). Therefore, strains with increased MIC values for amphotericin B, resulting from defects in ergosterol biosynthesis, are still impacted by the presence of *P. aeruginosa*.

To determine whether *P. aeruginosa* impacts the susceptibility of other *Candida* species to amphotericin B, we grew *P. aeruginosa* in dual-species biofilms with non-*albicans Candida* species for 24 h, and then treated the biofilms with 2 μg/mL amphotericin B. *C. dubliniensis* behaved similarly to *C. albicans*, being more susceptible to amphotericin B in the presence of *P. aeruginosa*. However, *C. tropicalis*, *C. parapsilosis*, *C. krusei* and *C. glabrata* still displayed significant culturability after treatment with amphotericin B in both mono- and dual-species biofilms (Figure 3c). The culturability of *C. tropicalis* was significantly reduced in the presence of *P. aeruginosa*, suggesting that the bacterium has some impact on *C. tropicalis*. On the other hand, the culturability of the other *Candida* species in dual-species biofilms was comparable to that of monospecies biofilms, indicating that *P. aeruginosa* has negligible impact on these *Candida* species. In addition, the amount of *P. aeruginosa* was not affected by the presence of the different *Candida* species, or the antifungal (Figure S1d).

### C. albicans mutants with reduced ergosterol content are more resistant to amphotericin B

**Figure 4.**
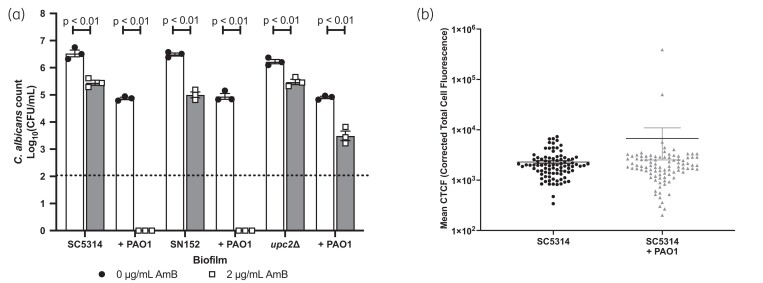
Deletion of *UPC2*, the key transcription factor regulating ergosterol biosynthesis, enhances the resistance of *C. albicans* to amphotericin B in the presence of *P. aeruginosa*. (a) *C. albicans* strains were grown in single- or dual-species biofilms for 24 h and then biofilms were treated with 2 μg/mL amphotericin B for 2 h. Dotted line represents the minimal level of detection of the assay. Data are the log_10_(mean) ± the SEM from three biological replicates. Data were analysed using two-way ANOVA and the Holm–Šidák multiple comparisons test. (b) Twenty-four-hour preformed biofilms were fixed with PFA, disrupted, and stained with filipin.

One of the main modes of action of amphotericin B is to bind ergosterol in the fungal membrane, forming pores resulting in fungal cell lysis. Genes involved in the synthesis of ergosterol are regulated by the Upc2 transcription factor.^25^ Therefore, the *upc2Δ* mutant synthesizes significantly less ergosterol than WT cells.^25^ To determine whether the increased susceptibility of *C. albicans* to amphotericin B in the presence of *P. aeruginosa* is dependent on ergosterol, we quantified the susceptibility of the *upc2Δ* mutant to amphotericin B in both mono- and dual-species biofilms. Deletion of *UPC2* attenuated the ability of *P. aeruginosa* to impact the susceptibility of *C. albicans* to the polyene, with significant fungal culturability observed in dual-species biofilms treated with amphotericin B (Figure 4a). Therefore, we hypothesized that *P. aeruginosa* increases the susceptibility of *C. albicans* to the polyene by increasing the amount of ergosterol in the fungal cell membrane. However, the levels of ergosterol were similar between mono- and dual-species biofilms (Figure 4b) suggesting that although ergosterol levels are important for the mode of action of amphotericin B, *P. aeruginosa* does not directly increase ergosterol incorporation into the fungal cell membrane.

### C. albicans undergoes a significant transcriptional response to the presence of P. aeruginosa

**Figure 5.**
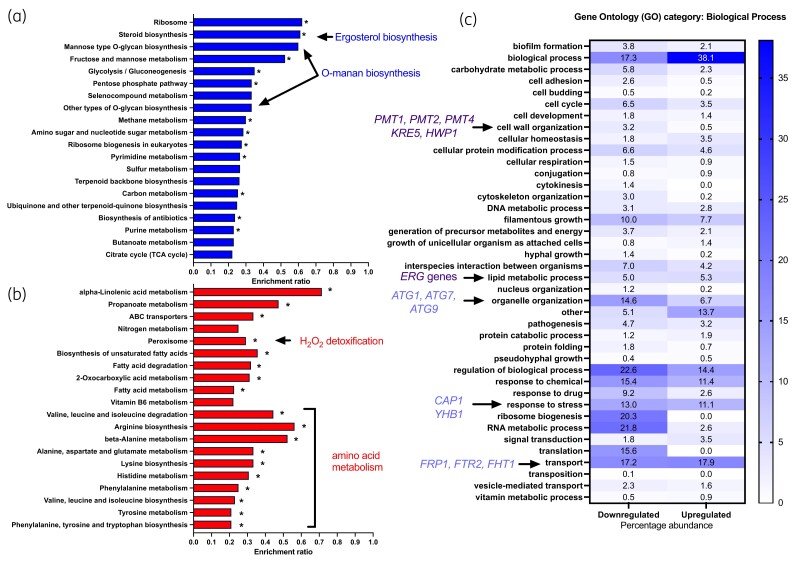
*C. albicans* undergoes a significant transcriptional response in dual-species biofilms. (a) KEGG enrichment analysis of pathways significantly down-regulated in dual-species biofilms. Ergosterol and *O*-mannan biosynthesis are highlighted. (b) KEGG enrichment analysis of pathways significantly up-regulated in dual-species biofilms. ROS detoxification and amino acid metabolism are highlighted. (c) GO slim term analysis of biological processes differentially regulated between single- and dual-species biofilms. Genes of interest in purple are significantly down-regulated, whereas genes in blue are significantly up-regulated (see text for details). This figure appears in colour in the online version of *JAC* and in black and white in the print version of *JAC*.

To elucidate how *P. aeruginosa* increases the susceptibility of *C. albicans* to amphotericin B, global transcriptional analysis was performed on *C. albicans* in both mono- and dual-species biofilms. The presence of *P. aeruginosa* resulted in a significant transcriptional response in *C. albicans,* with 431 genes being significantly up-regulated and 739 genes being significantly down-regulated (*P*adj < 0.05, and log_2_-fold cut-off  ≥1 or  ≤ −1) (Figure S2, File S1). In agreement with our ergosterol staining data, KEGG pathway enrichment analysis identified ergosterol biosynthesis to be among the biological processes that are significantly down-regulated in the dual-species biofilms, with 15 of the genes involved in ergosterol biosynthesis (*ERG1*, *ERG3–6*, *ERG9–13*, *ERG24*, *ERG25*, *ERG251*, *HMG1* and *MVD1*) being significantly down-regulated (Figure 5a). Therefore, the increased susceptibility of *C. albicans* to amphotericin B in the presence of *P. aeruginosa* is not due to increased ergosterol levels. From the significantly down-regulated genes, 24 were associated with fungal cell wall organization, with genes required for chitin and glucan synthesis (*CHT2*, *CHT3*, *ENG1*, *EXG2* and *KRE1*), filamentous growth (*ALS2–4*, *HWP1*, *HWP2*, *HYR1*, *SFL2*) and *O*-mannan biosynthesis (*PMT1*, *PMT2*, *PMT4* and *DMP1*) being down-regulated in dual-species biofilms. *P. aeruginosa* binds to *C. albicans* hyphae via *O*-mannans resulting in fungal cell death.^2,26^ Therefore, the down-regulation of these processes might prevent bacterial attachment and ensure fungal survival. Other processes that were significantly down-regulated included genes involved in ribosome biogenesis, translation and carbon metabolism (Figure 5a), suggesting that *C. albicans* is under stress in the dual-species biofilms. In agreement with this, genes involved in iron acquisition (*FRP1*, *FTR2*, *FHT1*) and amino acid metabolism were significantly up-regulated (Figure 5b, c), suggesting that there is nutrient competition in dual-species biofilms. Response to stress was also one of the GO terms enriched in our analysis (Figure 5c). To further understand the type of stress the bacterium imposes on *C. albicans*, we compared our transcriptional response with published transcriptional responses to heat shock, osmotic stress and oxidative stress.^17^ Among the conditions tested, genes differentially regulated in the dual-species biofilms showed a significant positive correlation with oxidative stress (Table S2), suggesting that *P. aeruginosa* might impose oxidative stress on *C. albicans*. In support of this, *CAP1*, a key transcription factor involved in the oxidative stress response, was significantly up-regulated in dual-species biofilms. KEGG and GO term analysis identified peroxisome function (*CTN3*, *ECI1*, *FAA21*, *FOX2*, *MDH1–3*, *OPT3*, *PEX5*, *PEX6*, *POT1*, *POX1–3*, *PXA1*, *PXA2*, *PXP2*, C2_00390C_A and CR_02570C_A), phagophore assembly (*APG7*, *ATG1*, *ATG9*, *AUT7*, C1_00860W_A, C1_03330C_A and C1_12150C_A) and oxidoreductase activity (51 genes including *GPX2*, *YHB1*, *YHB4*, *YHB5* and *SOD6*) to be significantly up-regulated in *C. albicans* in response to *P. aeruginosa* (Figure 5b, c). In addition to functioning in fatty acid oxidation, the fungal peroxisome is involved in detoxifying hydrogen peroxide, providing further evidence that *P. aeruginosa* may be imposing reactive oxygen stress on *C. albicans*. Up-regulation of genes associated with the phagophore suggests that autophagy has been induced. Taken together, these results suggest that *C. albicans* is under significant stress, likely oxidative stress, in the dual-species biofilms.

### P. aeruginosa increases the susceptibility of C. albicans to amphotericin B through the phenazine-mediated induction of ROS stress

**Figure 6.**
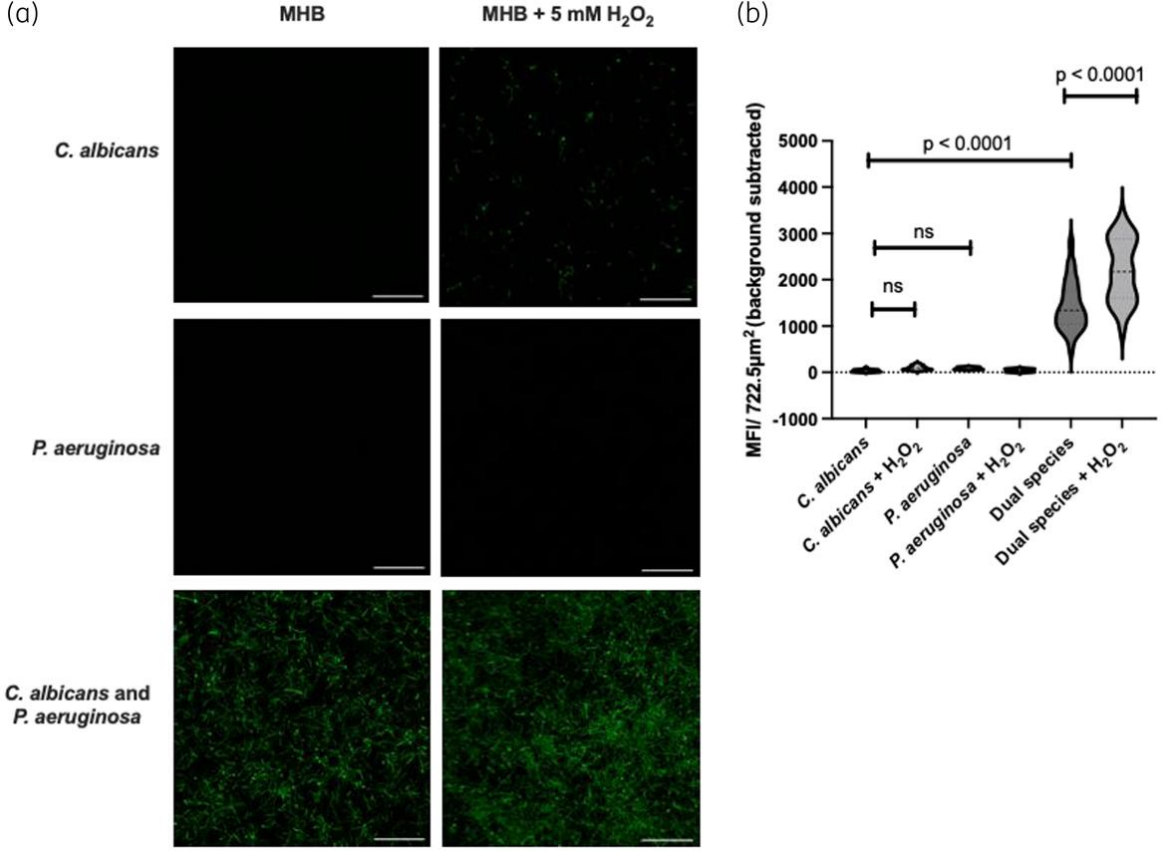
*C. albicans* is under ROS stress in dual-species biofilms. (a) Single- and dual-species biofilms were grown in MHB or MHB containing 5 mM H_2_O_2_ for 24 h. Biofilms were stained with H_2_DCFDA and imaged. (b) Biofilms were excited at 488 nm and emission captured between 500 and 550 nm. Mean fluorescence intensity was taken at three 850 × 850 μm regions per biofilm, with 9 biofilms assessed per condition (27 regions total), with unstained control biofilms to account for background fluorescence. Scale bar represents 100 μm. Data were analysed using one-way ANOVA and the Holm–Šidák multiple comparisons test. This figure appears in colour in the online version of *JAC* and in black and white in the print version of *JAC*.

Given that our transcriptomics data suggested that *C. albicans* might be under oxidative stress in the dual-species biofilms, we quantified the amount of ROS in single- and dual-species biofilms using the ROS-sensitive dye H_2_DCFDA in combination with confocal microscopy. Single-species biofilms of either *C. albicans* or *P. aeruginosa* exhibited minimal staining with H_2_DCFDA, suggesting that the levels of ROS in these biofilms was below detectable levels. As a positive control, single-species biofilms were grown in the presence of H_2_O_2_, a known ROS inducer. In the *C. albicans* single-species biofilms in response to 5 mM H_2_O_2_, some of the hyphal cells showed significant staining with H_2_DCFDA, suggesting that the intracellular ROS levels in these cells were higher than in the non-treated single-species biofilm cells. However, there was a significant increase in the number and fluorescence intensity of fungal cells stained with H_2_DCFDA in the untreated dual-species biofilms, confirming that the intracellular ROS levels in *C. albicans* were significantly increased in dual-species biofilms (Figure 6). Therefore, in the dual-species biofilms, *P. aeruginosa* exerts oxidative stress on *C. albicans* through the generation of intracellular ROS.

**Figure 7.**
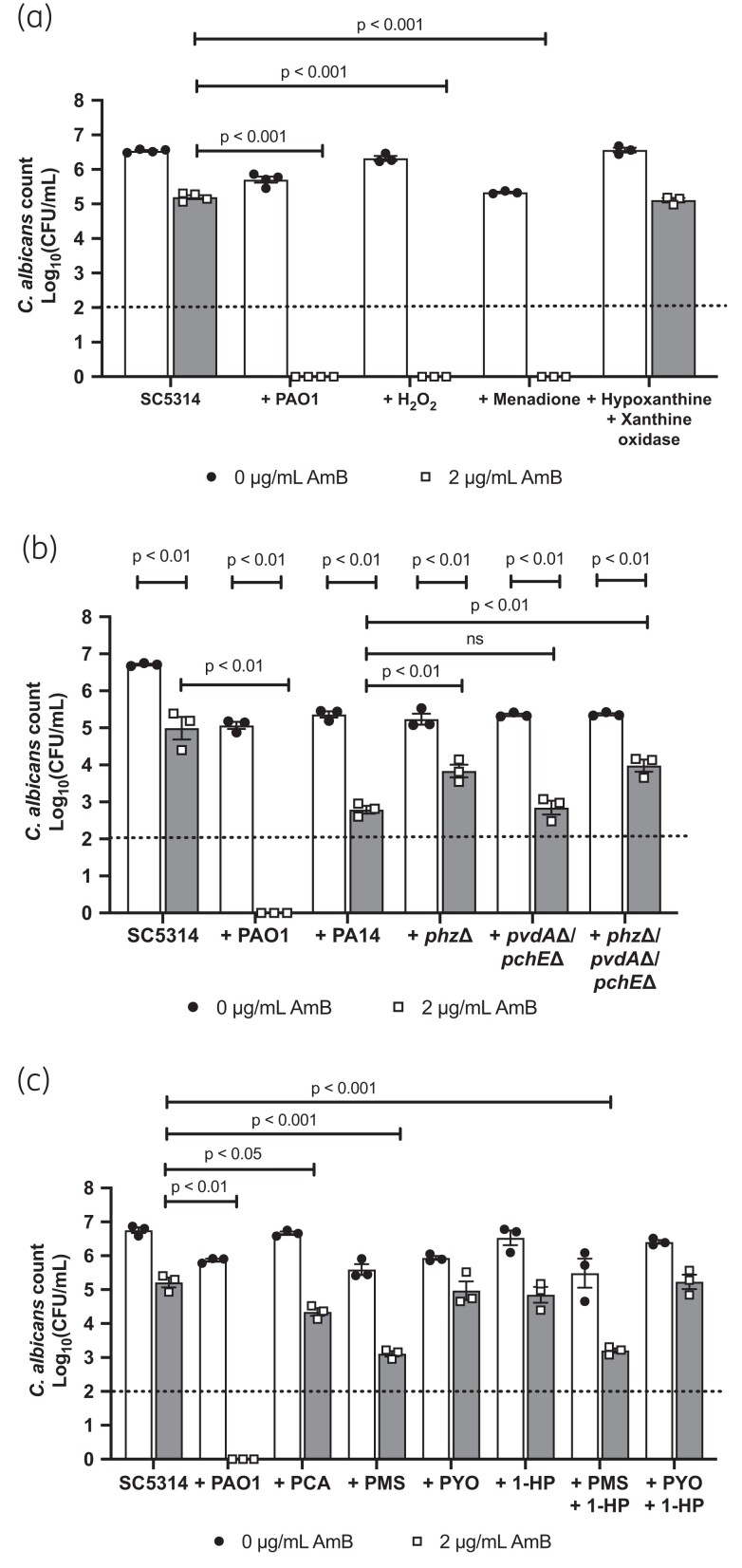
Increased susceptibility of *C. albicans* to amphotericin B is partially mediated by *P. aeruginosa* phenazines. (a) SC5314 single-species biofilms were grown in the presence of H_2_O_2_, menadione, and hypoxanthine and xanthine oxidase for 24 h, and then treated with 2 μg/mL amphotericin B for 2 h. (b) SC5314 was grown in single- or dual-species biofilms with *P. aeruginosa* strains defective in iron and phenazine biosynthesis. (c|) SC5314 single-species biofilms were grown in the presence of 40 μM purified phenazine compounds for 24 h, and then biofilms were treated with 2 μg/mL amphotericin B for 2 h. Data are the log_10_(mean) ± the SEM from three biological replicates. Dotted line represents the minimal level of detection of the assay. Data were analysed using two-way ANOVA and the Holm–Šidák multiple comparisons test. PCA, phenazine-1-carboxlic acid; PMS, phenazine methosulphate; PYO, pyocyanin; 1-HP,  1-hydroxyphenazine.

In addition to binding ergosterol and inducing cell lysis, amphotericin B has been shown to exert antifungal activity through the generation of ROS stress.^27^ At the same time, *P. aeruginosa* is also known to induce ROS stress in *C. albicans*.^5^ Therefore, we hypothesized that ROS induction by both *P. aeruginosa* and amphotericin B at the same time may result in enhanced antifungal activity. In support of this hypothesis, treatment of *C. albicans* monospecies biofilms with 10 mM H_2_O_2_ resulted in enhanced susceptibility of the fungus to amphotericin B, comparable to the effects observed in the presence of *P. aeruginosa* (Figure 7a). Similar effects were observed when *C. albicans* monospecies biofilms were established in the presence of menadione, an inducer of mitochondrial ROS. However, *C. albicans* biofilms grown in the presence of hypoxanthine and xanthine oxidase, which induce cellular ROS, did not increase the susceptibility of *C. albicans* to amphotericin B (Figure 7a). Therefore, it is likely that *P. aeruginosa* increases *C. albicans* susceptibility to the polyene through the induction of mitochondrial ROS.

*P. aeruginosa* has been shown to induce ROS stress in *C. albicans* through the secretion of phenazines.^5^ In agreement with phenazine-mediated ROS stress in *C. albicans*, deletion of genes involved in phenazine biosynthesis attenuated the ability of *P. aeruginosa* to enhance the susceptibility of *C. albicans* to amphotericin B (Figure 7b, Figure S1e). Addition of exogenous phenazines to *C. albicans* monospecies biofilms confirmed that phenazine methosulphate (PMS), and to a lesser extent, phenazine-1-carboxylic acid (PCA) mediated ROS stress and therefore enhanced *C. albicans* susceptibility to amphotericin B (Figure 7c).

### P. aeruginosa increases the susceptibility of C. albicans to amphotericin B by increasing mitochondrial ROS, while suppressing SOD2 expression

**Figure 8.**
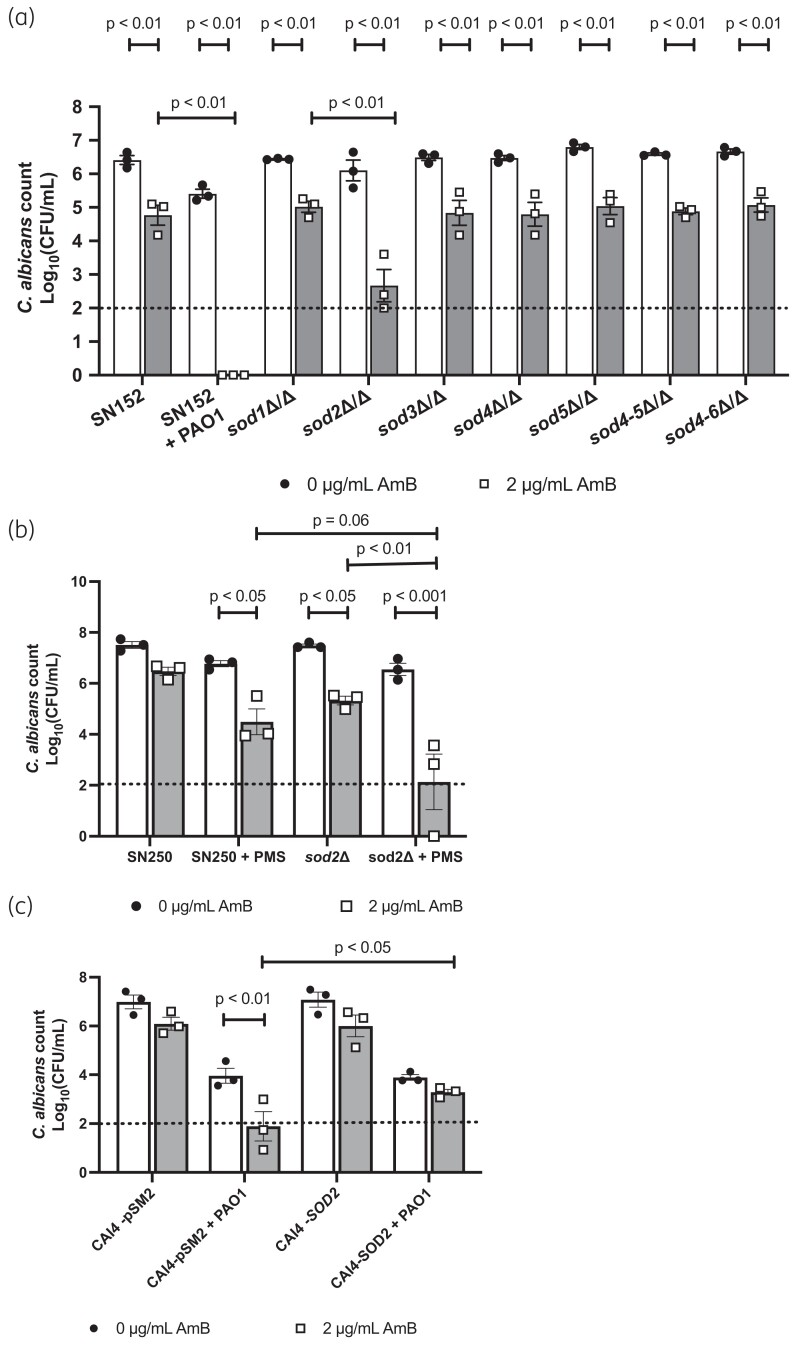
Overexpression of mitochondrial *SOD2* prevents hypersusceptibility of biofilms to amphotericin. (a) *C. albicans* SOD mutants were grown in single-species biofilms and then treated with 2 μg/mL amphotericin B for 2 h. (b) The parental control strain and the *sod2Δ* mutant were grown in single-species biofilms for 24 h and then treated with 2 μg/mL amphotericin B for 2 h. (c) The parental control strain and the *SOD2* overexpression (CAI4-*SOD2*) strain were grown in single- or dual-species biofilms for 24 h and then treated with 2 μg/mL amphotericin B for 2 h. Data are the log_10_(mean) ± the SEM from three biological replicates. Dotted line represents the minimal level of detection of the assay. Data were analysed using two-way ANOVA and the Holm–Šidák multiple comparisons test.

Superoxide radicals are removed through the actions of superoxide dismutase (Sod) enzymes. The *C. albicans* genome contains six SOD genes (*SOD1–6*), which vary in their subcellular localization. In response to ROS stress, many organisms up-regulate these enzymes to detoxify the superoxide radicals. However, analysis of our transcriptomic data confirmed that, in the presence of *P. aeruginosa*, *SOD1–5* were down-regulated in *C. albicans*, whereas *SOD6* was up-regulated (Table 1). Sod2 is a mitochondrial Sod enzyme, essential for the detoxification of mitochondrial ROS. Previous transcriptional studies confirm that, in response to oxidative stress, *SOD2* is significantly up-regulated to detoxify ROS, and is usually up-regulated in response to amphotericin B treatment.^28,29^ Given that both phenazines and amphotericin B target mitochondrial ROS production, and that menadione mimics the effects of *P. aeruginosa* on *C. albicans* susceptibility to amphotericin B, *P. aeruginosa*-induced repression of *SOD2* could enhance ROS toxicity. In agreement with this, deletion of *SOD2* resulted in significantly increased *C. albicans* susceptibility to amphotericin B in the absence of *P. aeruginosa,* whereas deletion of *SOD1* and *SOD3–6* had minimal effect on amphotericin B susceptibility (Figure 8a). Furthermore, addition of exogenous PMS to *sod2Δ* monospecies biofilms further enhanced the susceptibility of *C. albicans* to amphotericin B (Figure 8b). To determine whether *P. aeruginosa*-dependent suppression of *SOD2* was the driving force behind the increased susceptibility of *C. albicans* to amphotericin B, we expressed *SOD2* from a constitutive promoter and reassessed *C. albicans* viability upon amphotericin B treatment. Constitutive expression of *SOD2* resulted in increased resistance of *C. albicans* to amphotericin B in the presence of *P. aeruginosa* (Figure 8c), confirming that *P. aeruginosa*-mediated repression of *SOD2* expression contributes to the enhanced susceptibility of *C. albicans* to amphotericin B in dual-species biofilms.

**Table 1.** Expression of superoxide dismutase genes

ORF	Gene	Gene product	Fold change (log_2_)	*P*adj
19.2060	*SOD5*	Cu-containing Sod	−2.97	5.63 × 10^−9^
19.3340	*SOD2*	Mitochondrial Mn-containing Sod	−1.85	5.29 × 10^−17^
19.2770.1	*SOD1*	Cytosolic Cu- and Zn-containing Sod	−1.36	1.11 × 10^−5^
19.2062	*SOD4*	Cu-containing Sod	−0.59	3.04 × 10^−3^
19.7111.1	*SOD3*	Cytosolic Mn-containing Sod	−0.54	4.10 × 10^−1^
19.6229	*CAT1*	Catalase	0.43	1.54 × 10^−1^
19.2108	*SOD6*	Cell-surface Cu-containing Sod	2.01	2.20 × 10^−15^

## Discussion

Antimicrobial resistance is a global threat, with MDR strains frequently isolated in the clinic. It is now estimated that, by 2050, 10 million people per year will die as a result of antimicrobial-resistant infections.^30^ Although not as frequently mentioned as antibiotic resistance, antifungal resistance places a huge burden on patients and healthcare providers. This is exemplified by the isolation of MDR strains of *Candida auris*, with several strains being resistant to the three major classes of antifungals.^31^ As a result, the WHO and the CDC have declared antimicrobial-resistant *Candida* infections a global health threat. Therefore, there is an urgent need for the development of new classes of antifungal drugs, or for increased efficacy of existing treatment options. Here, we have shown that *P. aeruginosa* enhances the susceptibility of *C. albicans* to amphotericin B through phenazine-dependent induction of mitochondrial ROS stress in conjunction with the suppression of ROS-detoxifying enzymes.

Owing to the nephrotoxicity of amphotericin B, it is usually used as a last-resort treatment. However, liposomal formulations have greatly reduced the toxicity of the drug. Amphotericin B targets ergosterol in the fungal membrane, impairing membrane integrity, and has been shown to induce mitochondrial ROS stress.^27^ In agreement with this, the *C. albicans sod2Δ* mutant was more susceptible to amphotericin B than the deletion of genes encoding the other superoxide dismutases. *P. aeruginosa* has been shown to induce ROS stress in *C. albicans* through the secretion of phenazine compounds.^32^ Only PMS, a synthetic analogue of 5-methyl phenazine-1-carboxylic acid betaine (5MPCA), was able to increase the susceptibility of *C. albicans* to amphotericin B. *P. aeruginosa* secretes 5MPCA during dual-species growth with *C. albicans*,^33^ but not during monoculture,^34^ which may explain why culture supernatants from *P. aeruginosa* monocultures did not affect the susceptibility of *C. albicans* to amphotericin B. However, supplementation of *C. albicans* monospecies biofilms with PMS did not fully recapitulate the presence of the bacterium, suggesting that additional factors are required. 5MPCA can be modified by amino acids including arginine to form red pigmented proteins.^32^ Our transcriptional analysis identified amino acid biosynthesis, particularly arginine biosynthesis, to be significantly up-regulated in dual-species biofilms. Therefore, increases in amino acid biosynthesis might increase the bioactivity of the phenazine in dual-species biofilms. Supplementation of *sod2Δ* biofilms with PMS has a greater impact on the susceptibility of *C. albicans* to the polyene. *SOD2* is usually up-regulated in the presence of amphotericin B and other inducers of mitochondrial ROS stress.^28,29^ However, transcriptional analysis confirmed that, in the presence of *P. aeruginosa*, *SOD2* and several other detoxifying enzymes were down-regulated. Therefore, it would appear that *P. aeruginosa* may simultaneously induce ROS stress while down-regulating *SOD2* expression, which likely results in the stress surpassing the capacity of the detoxification system, thus leading to cell death.

*P. aeruginosa* is highly genetically variable.^35^ Although treatment of dual-species biofilms containing PAO1 with amphotericin B resulted in minimal fungal survival after 2 h, biofilms containing PA14 and *P. aeruginosa* clinical isolates required a longer incubation with the drug to completely eradicate the fungus (although a significant reduction in fungal culturability was observed after 2 h). Although all *P. aeruginosa* species produce 5MPCA, the production of the phenazine intermediate is variable between isolates.^33^ Therefore, it is likely that, in this assay, the species produce 5MPCA at lower levels compared with the PAO1 strain, which may explain why extended incubation times are required to fully eradicate the fungus.

Other classes of antifungals also induce ROS production in *C. albicans* as part of the mode of action,^36^ suggesting that *P. aeruginosa* should also increase the susceptibility of the fungus to other antifungals. Fluconazole targets ergosterol biosynthesis, reducing ergosterol levels in the fungal membrane, and resulting in the accumulation of toxic sterols.^37,38^ As a result, fluconazole is fungistatic against *C. albicans.* However, *in vivo P. aeruginosa* has been shown to make fluconazole fungicidal toward *C. albicans*.^39^ Yet, in our biofilm assay, fluconazole had minimal effect on the culturability of *C. albicans*, whether in the absence or presence of the bacterium. However, glucans in the ECM sequester fluconazole, preventing it from penetrating the biofilm.^40^ Therefore, it is likely that, as fluconazole is added to mature biofilms, the glucan component of the ECM sequesters the drug, preventing it from reaching the fungal cells.

Although *C. albicans* is still the main species causing candidiasis, the incidence of infections involving non-*albicans Candida* species is increasing.^41^ Therefore, it is important for antifungal therapies to be effective across all *Candida* species. *P. aeruginosa* enhanced the susceptibility of *C. dubliniensis* to amphotericin B, similarly to *C. albicans*, which is not surprising, as *C. dubliniensis* is the most closely related species to *C. albicans*. Amphotericin B had a negligible effect on the viability of this isolate of *C. krusei* both in the presence or absence of *P. aeruginosa*, suggesting that this isolate is resistant to the polyene. Estimates suggest that up to 15% of *C. krusei* isolates are resistant to amphotericin B owing to reduced ergosterol content in the cell membrane.^42^ Despite the other *Candida* species being susceptible to the polyene, their susceptibility was not significantly enhanced in the presence of *P. aeruginosa.* The interactions between *P. aeruginosa* and non-*albicans Candida* species have not been as extensively studied as *C. albicans*. Therefore, it is possible that either 5MPCA does not induce ROS stress, that the bacterium is not able to down-regulate the ROS detoxifying enzymes in these species, or that these species can tolerate greater levels of ROS than *C. albicans*.

In the clinical setting approximately 80% of infections are due to the formation of biofilms, which are highly antimicrobial resistant due to the up-regulation of efflux pumps and the ECM providing protection from the environment.^43–45^ Therefore, *in vivo*, much higher concentrations of drug are required to eradicate the infection, but cannot always be achieved. Our data confirm that, in a mixed-species biofilm, *C. albicans* is more susceptible to amphotericin B than in monospecies biofilms. Given that *in vivo* biofilms are normally composed of multiple species, especially in traumatic wounds and in the cystic fibrosis lung where *C. albicans* and *P. aeruginosa* are frequently co-isolated, this suggests that *C. albicans* might be more susceptible to amphotericin B than the other classes of antifungal drugs. However, confirmation of our simple *in vitro* biofilm results in more clinically relevant biofilm models and *in vivo* infection settings is required, to confirm whether this enhanced susceptibility to amphotericin B is maintained. Given that we, and others, have shown that *C. albicans* promotes drug tolerance in multiple bacterial species,^12,14,15^ treatment of polymicrobial biofilms with an initial course of antifungal, prior to the initiation of antibacterial treatment, may help to overcome some of the antimicrobial resistance observed. However, this requires further investigation.

## Supplementary Material

dkad228_Supplementary_Data

## Supplementary data

Figures S1 and S2, Tables S1 and S2 and File S1 are available as Supplementary data at *JAC* Online.
