# Supplementary material for: Pseudomonas aeruginosa increases the susceptibility of Candida albicans to amphotericin B in dual-species biofilms
Source: J Antimicrob Chemother. 2023 Jul 31;78(9):2228–41. doi: 10.1093/jac/dkad228 (PMC10477122; doi:10.1093/jac/dkad228)
Supplement: dkad228_Supplementary_Data [file dkad228_supplementary_data.zip › supplemental_data_CLEAN.docx]

**Figure S1. Treatment does not impair *P. aeruginosa* culturability** **A)** Culturability of *P. aeruginosa* in response to amphotericin B. **B)** Culturability of *P. aeruginosa* in response to fluconazole. C) Culturability of *P. aeruginosa* clinical isolates after 2 or 18 hr treatment with amphotericin B. **D)** PAO1 culturability in the presence of other non-*albicans* strains of *Candida*. **E)** Culturability of PA14 and siderophore and phenazine mutants after 2 hrs of amphotericin B treatment.

**Figure S2. Transcriptional profiling of *C. albicans* in single- and dual-species biofilms. A)** PCA plot. **B)** Volcano plot of significantly differentially regulated genes (p <0.05 and log_2_-fold change ≥ 1).

**Table S1. Strains used in the study.**

| **Strain** | **Genotype** | **Source** |
| --- | --- | --- |
| ***Candida* strains** | | |
| SC5314 | Wild type | ^46^ |
| SVS002B | Clinical isolate | Gordon Ramage, Glasgow University |
| SVS006B | Clinical isolate | Gordon Ramage, Glasgow University |
| SVS021B | Clinical isolate | Gordon Ramage, Glasgow University |
| SVS0036A | Clinical isolate | Gordon Ramage, Glasgow University |
| SVS051A | Clinical isolate | Gordon Ramage, Glasgow University |
| DSY4 | *erg6*∆::FRT/*erg6*∆::*SAT1*::*FRT* | ^47^ |
| *Candida dubliniensis* | Wild type | ^48^ |
| *Candida tropicalis* | Type strain | ATCCMYA-3404 |
| *Candida parapsilosis* | Type strain | ^49^ |
| *Candida krusei* | Clinical isolate | ^15^ |
| *Candida glabrata* | Type strain | ATCC2001 |
| SN152 | *arg4∆/arg4∆, leu2∆/leu2∆, his1∆/his1∆, URA3/ura3∆* | ^50^ |
| SN250 | *his1*Δ/*his1*Δ; *leu2*Δ/*leu2*Δ; *arg4*Δ/*arg4*Δ;  *URA3/ura3*Δ::imm434; *IRO1/iro1*Δ::*imm434* | ^50^ |
| *sod1Δ* | *his1Δ/his1Δ; leu2Δ/leu2Δ; arg4Δ/arg4Δ;*  *URA3/ura3Δ::imm434; IRO1/iro1Δ::imm434;*  *sod1Δ::C.dubliniensisHIS1/sod1Δ::C.maltosaLEU2* | ^51^ |
| *sod2Δ* | *his1Δ/his1Δ; leu2Δ/leu2Δ; arg4Δ/arg4Δ;*  *URA3/ura3Δ::imm434; IRO1/iro1Δ::imm434;*  *sod2Δ::C.dubliniensisHIS1/sod2Δ::C.maltosaLEU2* | ^51^ |
| *sod3Δ* | *his1Δ/his1Δ; leu2Δ/leu2Δ; arg4Δ/arg4Δ;*  *URA3/ura3Δ::imm434; IRO1/iro1Δ::imm434;*  *sod3Δ::C.dubliniensisHIS1/sod3Δ::C.maltosaLEU2* | ^51^ |
| *sod4Δ* | *his1Δ/his1Δ; leu2Δ/leu2Δ; arg4Δ/arg4Δ;*  *URA3/ura3Δ::imm434; IRO1/iro1Δ::imm434;*  *sod4Δ::C.dubliniensisHIS1/sod4Δ::C.maltosaLEU2* | ^51^ |
| *sod5Δ* | *his1Δ/his1Δ; leu2Δ/leu2Δ; arg4Δ/arg4Δ;*  *URA3/ura3Δ::imm434; IRO1/iro1Δ::imm434;*  *sod5Δ::C.dubliniensisHIS1/sod5Δ::C.maltosaLEU2* | ^51^ |
| *sod4-5Δ* | *his1Δ/his1Δ; leu2Δ/leu2Δ; arg4Δ/arg4Δ;*  *URA3/ura3Δ::imm434; IRO1/iro1Δ::imm434;*  *sod5Δ::C.dubliniensisHIS1/sod5Δ::C.maltosaLEU2; sod4∆::FRT/sod4∆::SAT1-FLP* | ^51^ |
| *sod4-6Δ* | *his1Δ/his1Δ; leu2Δ/leu2Δ; arg4Δ/arg4Δ;*  *URA3/ura3Δ::imm434; IRO1/iro1Δ::imm434;*  *sod5Δ::C.dubliniensisHIS1/sod5Δ::C.maltosaLEU2;*  *sod4∆::FRT/sod4∆::FRT; sod6∆::FRT/sod6∆::FRT* | ^51^ |
| CAI4-*SOD2* | *ura3Δ::imm434/ura3Δ::imm434*; pTEF2-SOD2 | This study |
| *upc2Δ* | *his1Δ/his1Δ; leu2Δ/leu2Δ; arg4Δ/arg4Δ;*  *URA3/ura3Δ::imm434; IRO1/iro1Δ::imm434;*  *upc2Δ::C.dubliniensisHIS1/upc2Δ::C.maltosaLEU2* | ^52^ |
| ***Pseudomonas aeruginosa strains*** | | |
| PAO1 | Wild-type | ATCC15692 |
| PA14 | Wild-type | ^53^ |
| CF (Midlands 1) | Clinical isolate | ^54^ |
| CF (Liverpool) | Clinical isolate | ^55^ |
| *phzΔ* | Deletions of operons *phzA1–G1* and  *phzA2–G2* | ^56^ |
| *pvdAΔ/pchEΔ* | Deletions of *pvdA* and *pchE* | ^57^ |
| *phzΔ/pvdAΔ/pchEΔ* | Deletions of *pvdA, pchE*, and operons  *phzA1–G1* and *phzA2–G2* | ^57^ |

**Table S2. Correlation of differentially regulated genes with stress responses**

| **Condition** | **Pearson’s correlation coefficient** | **P-value** |
| --- | --- | --- |
| oxidative stress 0 min | -0.02707543 | 0.4083 |
| oxidative stress 10 min | 0.3700462 | <2.2e-16 |
| oxidative stress 30 min | 0.4379898 | <2.2e-16 |
| oxidative stress 60 min | 0.2234399 | 4.82E-12 |
| heat stress 0 min | 0.04271131 | 0.1896 |
| heat stress 10 min | 0.267834 | < 2.2e-16 |
| heat stress 30 min | 0.2829612 | < 2.2e-16 |
| heat stress 60 min | 0.2216566 | 5.55E-12 |
| hyperosmotic stress 0 min | -0.05825235 | 0.07502 |
| hyperosmotic stress 10 min | 0.1306022 | 6.19E-05 |
| hyperosmotic stress 30 min | 0.05289894 | 0.106 |
| hyperosmotic stress 60 min | -0.1118003 | 0.0006153 |

**References**

46. Gillum AM, Tsay EYH, Kirsch DR. Isolation of the *Candida albicans* gene for orotidine-5′-phosphate decarboxylase by complementation of *S. cerevisiae* *ura3* and *E. coli* pyrF mutations. *Mol and Gen Genet* 1984; **198**: 179-82.

47. Dorsaz S, Snäkä T, Favre-Godal Q et al. Identification and Mode of Action of a Plant Natural Product Targeting Human Fungal Pathogens. *Antimicrob Agents Chemother* 2017; **61**.

48. Morschhäuser J, Ruhnke M, Michel S et al. Identification of CARE-2-negative *Candida albicans* isolates as *Candida dubliniensis*. *Mycoses* 1999; **42**: 29-32.

49. Zwolinska-Wcislo M, Budak A, Trojanowska D et al. Fungal colonization of the stomach and its clinical relevance. *Mycoses* 1998; **41**: 327-34.

50. Noble SM, Johnson AD. Strains and Strategies for Large-Scale Gene Deletion Studies of the Diploid Human Fungal Pathogen *Candida albicans*. *Eukaryotic Cell* 2005; **4**: 298-309.

51. Frohner IE, Bourgeois C, Yatsyk K et al. *Candida albicans* cell surface superoxide dismutases degrade host-derived reactive oxygen species to escape innate immune surveillance. *Mol Microbiol* 2009; **71**: 240-52.

52. Noble SM, French S, Kohn LA et al. Systematic screens of a *Candida albicans* homozygous deletion library decouple morphogenetic switching and pathogenicity. *Nature genetics* 2010; **42**: 590-8.

53. Rahme LG, Stevens EJ, Wolfort SF et al. Common virulence factors for bacterial pathogenicity in plants and animals. *Science* 1995; **268**: 1899-902.

54. Scott FW, Pitt TL. Identification and characterization of transmissible *Pseudomonas aeruginosa* strains in cystic fibrosis patients in England and Wales. *J Med Microbiol* 2004; **53**: 609-15.

55. Cheng K, Smyth RL, Govan JR et al. Spread of beta-lactam-resistant *Pseudomonas aeruginosa* in a cystic fibrosis clinic. *Lancet* 1996; **348**: 639-42.

56. Dietrich LEP, Price-Whelan A, Petersen A et al. The phenazine pyocyanin is a terminal signalling factor in the quorum sensing network of *Pseudomonas aeruginosa*. *Molecular Microbiology* 2006; **61**: 1308-21.

57. Wang Y, Wilks JC, Danhorn T et al. Phenazine-1-carboxylic acid promotes bacterial biofilm development via ferrous iron acquisition. *J Bacteriol* 2011; **193**: 3606-17.
